# Stimulation of the proliferation of human normal esophageal epithelial cells by fumonisin B_1_ and its mechanism

**DOI:** 10.3892/etm.2013.1364

**Published:** 2013-10-25

**Authors:** SHAO-KANG WANG, TING-TING WANG, GUI-LING HUANG, RUO-FU SHI, LI-GANG YANG, GUI-JU SUN

**Affiliations:** 1 Key Laboratory of Environmental Medicine and Engineering, Ministry of Education, School of Public Health, Southeast University, Nanjing, Jiangsu 210009, P.R. China; 2 Department of Nutrition and Food Hygiene, School of Public Health, Southeast University, Nanjing, Jiangsu 210009, P.R. China

**Keywords:** fumonisin B_1_, normal human esophageal epithelial cells, proliferation, cell cycle, apoptosis

## Abstract

Previous epidemiological studies have demonstrated a correlation between fumonisin B_1_ (FB_1_) and human esophageal cancer in China, Iran and South Africa. The purpose of this study was to investigate the effects of FB_1_ on the proliferation, cell-cycle and apoptosis of normal human esophageal epithelial cells (HEECs) and to explore the molecular mechanisms of these effects. The proliferation of HEECs treated with FB_1_ was assessed using a colorimetric assay, while analyses of the cell cycle and apoptosis were performed using flow cytometry and the measurement of the protein expressions of genes associated with the cell cycle was conducted using western blotting. The results showed that FB_1_ stimulated the proliferation of HEECs, decreased the percentage of cells in the G0/G1 phase and reduced apoptosis. The western blotting results showed that FB_1_ significantly increased the protein expression of cyclin D1 and significantly decreased the protein expression of cyclin E, p21 and p27. The results indicated that FB_1_ stimulated the proliferation of HEECs by affecting the cell cycle and apoptosis. This mechanism was associated with changes in cyclin D1, cyclin E, p21 and p27 expression.

## Introduction

Fumonisins are toxic metabolites produced predominantly by *Fusarium verticillioides*, one of the most common molds present globally on maize and other agricultural products (1–3). Areas of low and high esophageal cancer incidence in the former Transkei region of South Africa have been shown to correlate with low and high levels of fumonisin-contaminated home-grown maize, respectively (4). Alizadeh *et al*(5) detected high levels of fumonisin B_1_ (FB_1_) contamination in corn and rice samples from the Golestan province of Iran, with a significant positive correlation between the FB_1_ contamination in rice and the risk of esophageal cancer (5). The high contamination rates of FB_1_, the most common fumonisin, which are noted in food from Huaian where incidences of esophageal cancer are amongst the highest in China, suggest a possible contributing role of FB_1_ in human esophageal carcinogenesis (6). It has been proposed that FB_1_ may exhibit carcinogenicity in humans and FB_1_ is, at present, considered to be a possible carcinogen to humans. A number of studies have investigated the toxicity of FB_1_ in human cell lines to provide more information concerning the effects of FB_1_ in humans (7–10). FB_1_ was shown to inhibit clonal expansion in human keratinocytes and proliferation in human fibroblasts (7), in addition to causing DNA damage of an apoptotic type in human fibroblasts (8). Furthermore, FB_1_ has been demonstrated to induce apoptosis in human proximal tubule-derived cells (IHKE cells) (9) and to cause oxidative stress in the human intestinal cell line Caco-2 (10).

Although the toxic effects of FB_1_ on mammalian cells have been studied extensively, the carcinogenicity of FB_1_ on normal human esophageal epithelial cells (HEECs), and the possible mechanism underlying the effects, have yet to be elucidated. In recent years, numerous studies have shown that FB_1_ exerts significant effects on the cell cycle in certain cells (11,12). Cell cycle progression is controlled by cyclin-dependent kinases (CDKs) and cyclins (13,14). Cyclins D1 and E are necessary for entry into the S phase. CDK inhibitors, such as p16, p21 and p27, bind CDK-cyclin complexes and inhibit CDK activity (15,16).

In this study the effects of FB_1_ on the proliferation, cell cycle and apoptosis of HEECs were investigated, in addition to the expression of molecular markers of the cell cycle genes cyclins D1 and E, p16, p21 and p27 in HEECs. Furthermore, the potential esophageal carcinogenicity of FB_1_ in humans was examined.

## Materials and methods

### Materials

FB_1_, propidium iodide (PI), dimethylsulfoxide (DMSO), ethidium bromide (EB) and diethyl pyrocarbonate (DEPC) were purchased from Sigma (St. Louis, MO, USA), while RPMI-1640 and trypsin were obtained from Gibco (Grand Island, NY, USA). A stock solution of FB_1_ for cellular assays was prepared in phosphate-buffered saline (PBS) and then diluted in the optimal medium (≤10 μl/ml). Ethylenediaminetetraacetic acid (EDTA) was purchased from Calbiochem (San Diego, CA, USA) and fetal bovine serum (FBS) was purchased from Hangzhou Sijiqing Biological Engineering Material Co., Ltd. (Hangzhou, China). The reagents and membranes used for the protein assays, electrophoresis and western blotting were obtained from Bio-Rad (Hercules, CA, USA).

### Cell culture

The HEECs were obtained from Wuhan PriCells Biomedical Technology Co., Ltd. (Wuhan, China). The cell line is derived from the esophageal tissues of a 4-month-old female aborted fetus. Immunocytochemistry demonstrated the expression of cytokeratin, confirming the epithelial origin of the cells. The cells were cultured in RPMI-1640 medium supplemented with 5% FBS at 37ºC/95% humidity/5% CO_2_ in a humidified incubator. The growth of the cultured cells was observed daily using an inverted microscope (Olympus IX51; Olympus Corporation, Tokyo, Japan), and the RPMI-1640 medium was changed according to its color every 2–3 days.

### Cell viability assay

The HEECs (1×10^4^ cells/100 μl/well) were seeded in 96-well plates with 100 μl culture medium containing 5% FBS, and were incubated for 24 h to allow the cells to attach to the bottom of the plate. The cells were then incubated with 5, 10, 20 and 40 μmol/l FB_1_, respectively, for 24, 48, 72 and 96 h. Following the addition of 100 μl MTT (5 mg/ml in PBS) to the culture medium, the cells were incubated for 4 h at 37ºC with 5% CO_2_ in a humidified atmosphere. The medium was subsequently aspirated and the cells were suspended in 150 μl DMSO. The absorption was measured at 490 nm with a Mithras LB 940 Multimode Microplate Reader (Berthold Technologies GmbH, Bad Wildbad, Germany). The cell proliferation was calculated as follows: [optical density (OD) of the experimental sample/OD of the control] ×100. The experiment and assay were repeated at least three times.

### Harvesting cells

HEECs in the logarithmic growth phase were plated at a density of 1×10^5^ cells/ml in 50-cm^2^ culture flasks and allowed to grow in 4 ml culture medium. Following cell attachment, the culture medium was poured away and the cells were treated with FB_1_ (5, 10, 20 and 40 μmol/l) for 72 h. The cells were then trypsinized and collected for cell cycle and apoptosis analyses, or washed twice with ice-cold PBS and removed from the surface of the flask using a rubber scraper for western blot analysis.

### Cell cycle and apoptosis analyses

The cell cycle phase and apoptosis were examined using a Becton-Dickinson FACSCalibur Flow Cytometer (BD Biosciences, Franklin Lakes, NJ, USA). The cells were stained with Vindelov’s reagent (40 mM Tris, pH 7.6; 100 mM NaCl; 10 mg RNase A/ml; 7.5% PI and 0.1% Nonidet P-40), and data from 10,000 cells were collected for each data file. The experiment and assay were repeated three times.

### Western blotting

The cells detached by scraping were centrifuged for 10 min at 16,000 × g, 4ºC. The cell pellets were lysed in Mammalian Cell Protein Extraction Reagent [20 mM Tris, 0.1% sodium dodecyl sulfate (SDS), 1% Triton X-100, 1% sodium deoxycholate, pH 7.4] and Mammalian Protease Inhibitor Mixture. The supernatant was collected following centrifugation for 20 min at 10,000 × g, 4ºC. The protein concentration was assessed using a Pierce^®^ bicinchoninic acid (BCA) protein assay kit (Thermo Fisher Scientific Inc., Rockford, IL, USA) and lysates (30 μl total protein) were separated using SDS-polyacrylamide gel electrophoresis (PAGE). Following electrophoresis, the proteins were transferred onto polyvinylidene fluoride membranes. The membranes were then blocked in Tris-buffered saline with 0.1% Tween-20 (TBST), containing 5% non-fat dry milk, for 1 h at room temperature and incubated with primary antibody at 4ºC overnight. The membranes were then incubated at room temperature with horseradish peroxidase (HRP)-conjugated mouse or rabbit immunoglobulin G (IgG). The antibodies used in the western blotting and their dilutions are shown in Table I. After washing with TBST, incubation with West Pico Chemiluminescent Substrate (Thermo Fisher Scientific Inc., Waltham, MA, USA) and detection using Kodak *In-Vivo* Imaging systems (Carestream Health Inc., Rochester, NY, USA) enabled the visualization of the proteins, which were then quantified by strip densitometry. Actin was used as an internal control.

### Statistical analysis

The data are expressed as the mean ± standard deviation. Statistical analysis of the data was performed by one-way analysis of variance (ANOVA) with the SPSS statistical software package (version 13.0; SPSS, Inc., Chicago, IL, USA). Differences among the groups were evaluated using the parametric Least Significant Difference (LSD) test and differences between the experimental and the negative control groups were evaluated using a Dunnett’s test. P<0.05 was considered to indicate a statistically significant difference.

## Results

### Effect of FB_1_ on the proliferation of HEECs

**Figure 1:**
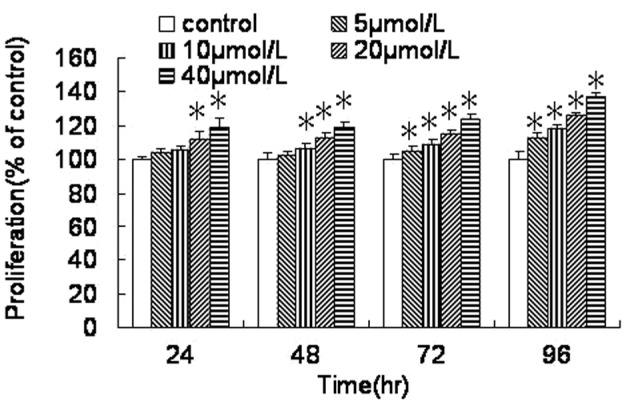
Effect of fumonisin B_1_ (FB_1_) on the growth of normal human esophageal epithelial cells (HEECs). The HEECs were treated with various concentrations of FB_1_ (5, 10, 20 and 40 μmol/l) for 24, 48, 72 and 96 h. Cell growth was measured using the MTT assay and the proliferation of cells was calculated. Data are expressed as the mean ± standard deviation from three independent experiments, each performed in triplicate. ^*^P<0.05, compared with the control.

As shown in Fig. 1, the proliferation of the HEECs was increased compared with that in the control group following treatment with FB_1_ for 24, 48, 72 and 96 h. The HEEC proliferation was significantly stimulated by treatment with FB_1_ (20 and 40 μmol/l) for 24 h, with FB_1_ (10, 20 and 40 μmol/l) for 48 h and with FB_1_ (5, 10, 20 and 40 μmol/l) for 72 and 96 h. Compared with the control group (100%), the proliferation of the HEECs was significantly increased to ~137.3±2.0% following treatment with 40 μmol/l FB_1_ for 96 h. The proliferation of the HEECs induced by treatment with 5, 10, 20 and 40 μmol/l FB_1_ for 72 h was increased significantly to 105.0±3.3%, 109.0±2.9%, 115.4±2.2% and 123.4±3.4%, respectively.

### Effect of FB_1_ on the cell cycle and apoptosis of HEECs

**Figure 2:**
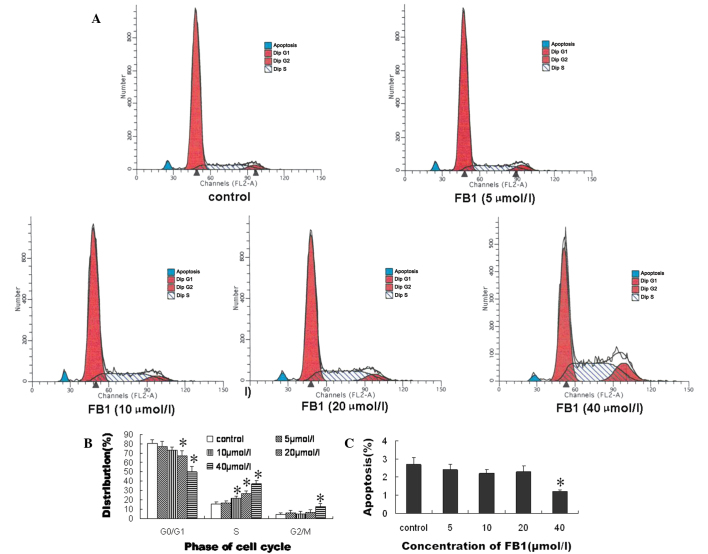
Effect of fumonisin B_1_ (FB_1_) on the cell cycle distribution and apoptosis in normal human esophageal epithelial cells (HEECs). The cells were incubated with various concentrations of FB_1_ for 72 h. The cell cycle distribution and apoptosis were analyzed using propidium iodide (PI) staining and the relative percentages were calculated. (A) One of representative image of three independent experiments is shown. (B) Results showing the cell cycle distribution percentages. (C) Results showing the cell apoptosis percentages. Data are expressed as the mean ± standard deviation from three independent experiments, each performed in triplicate. ^*^P<0.05, compared with the control.

In order to explore the mechanism by which FB_1_ affected the proliferation of the HEECs, the percentages of HEECs in the different phases of the cell cycle and cell apoptosis were assessed by flow cytometry. As shown in Fig. 2, the cell cycle progression of the HEECs was blocked in the S phase by treatment with FB_1_ in a dose-dependent manner, and was blocked in the G2/M phase by treatment with 40 μmol/l FB_1_. The percentages of HEECs in the G0/G1 phase were 67.1±4.7 and 49.8±5.9%, respectively, following treatment with 20 and 40 μmol/l FB_1_ (Fig. 2B). Compared with the control, the percentage of HEECs undergoing apoptosis was significantly decreased by treatment with 40 μmol/l FB_1_ (Fig. 2C).

### Effect of FB_1_ on the protein expression of genes involved in the cell-cycle in HEECs

**Figure 3:**
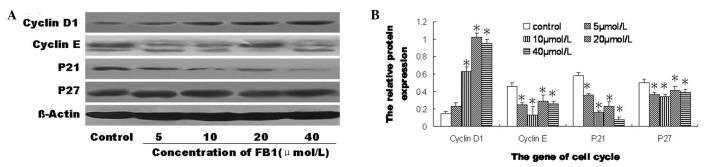
Effect of fumonisin B_1_ (FB_1_) on the protein expression of genes involved in the cell cycle in normal human esophageal epithelial cells (HEECs). Cells were treated with the indicated concentrations of FB_1_ for 72 h. The protein expression levels of cyclins D1 and E, p16, p21 and p27 in the HEECs were measured using western blotting. The HEECs treated with FB_1_ and the control cells were negative for the protein expression of p16. (A) Examples of western blotting showing the protein expression of genes involved in the cell cycle in HEECs in response to various concentrations of FB_1_. (B) The results showed that FB_1_ significantly upregulated the protein expression of cyclin D1 and significantly downregulated the protein expression of cyclin E, p21 and p27 in HEECs. Data are expressed as the mean ± standard deviation from three independent experiments, each performed in triplicate. ^*^P<0.05, compared with the control.

The protein expression levels of cyclins D1 and E, p21 and p27 in the HEECs were significantly changed by treatment with FB_1_ for 72 h compared with the levels in the control (Fig. 3). However, the HEECs treated with FB_1_ and the control cells were negative for the protein expression of p16 (data not shown). Compared with the control, the protein expression of cyclin D1 was significantly increased by treatment with 10, 20 and 40 μmol/l FB_1_, and the protein expression levels of cyclin E, p21 and p27 were significantly decreased by treatment with 5, 10, 20 and 40 μmol/l FB_1_ (Fig. 3B). These results showed that FB_1_ significantly increased the protein expression level of cyclin D1 and significantly decreased the protein expression levels of cyclin E, p21 and p27 in the HEECs.

## Discussion

Mycotoxins pose a health hazard to animals and humans through commonly contaminated staple food grains. FB_1_ is a cytotoxic and carcinogenic mycotoxin produced by *Fusarium verticillioides*, which causes porcine pulmonary edema and equine leukoencephalomalacia and has been implicated in the etiology of esophageal cancer in the Transkei, South Africa (17). Previous epidemiological surveys in China have revealed that FB_1_ is associated with the occurrence of human esophageal cancer (6,18). The International Agency for Research on Cancer has classified FB_1_ as a class 2B carcinogen, a probable human carcinogen (19). In order to investigate the effect of FB_1_ on the human esophagus, HEECs derived from a normal human esophagus were tested in the present study.

The results demonstrated that cell proliferation was stimulated in HEECs treated with FB_1_. Similarly, lower concentrations of mycotoxins (aflatoxin B1, FB_1_, deoxynivalenol and nivalenol) have been shown to enhance cellular proliferation, with the effect being more pronounced in human than in porcine lymphocytes (20). In another study, the stimuli for cell proliferation were introduced 7 days subsequent to the start of the administration of FB_1_, by gavage with different daily doses ranging from 0.14 to 3.5 mg FB_1_/100 g body weight, while cancer initiation was effected over a period of 14 days of FB_1_ treatment in the rat liver (21). The results of the present study demonstrated that the maximum proliferation of the HEECs was induced by treatment with 40 μmol/l FB_1_ for 96 h (137.3±2.0%). Furthermore, the proliferation of the HEECs induced by treatment with 5, 10, 20 and 40 μmol/l FB_1_ for 72 h was increased significantly to 105.0±3.3, 109.0±2.9, 115.4±2.2 and 123.4±3.4%, respectively. However, antiproliferative effects of FB_1_ have been observed in human hepatoma cells (22) and in swine peripheral blood mononuclear cells (23). In addition, Fornelli *et al (*24) showed an inhibition of proliferation in SF-9 cells treated with FB_1_ of ~20%. The varying effects of the FB_1_ on cell proliferation are dependent on the dose of the mycotoxins and the types of cells.

Compared with the control, treatment with 40 μmol/l FB_1_ for 72 h decreased the percentage of cells in the G0/G1 phase and increased the percentage of cells in the S and G2/M phases, in addition to significantly decreasing the percentage of HEECs undergoing apoptosis. In a previous study, flow cytometric and morphological analyses showed that FB_1_ lowered the marked apoptosis induced by prostaglandins, particularly prostaglandin A2, in esophageal carcinoma (WHCO3) cells. However, in combination with arachidonic acid and prostaglandins E2 and A2, FB_1_ increased the number of G2/M cells (25). In a different study, the results showed that the number of C6 glioma cells in the S phase decreased significantly compared with the control, from 18.7±2.5 to 8.1±1.1% for 9 μmol/l FB_1_, and the number of cells in the G2/M phase increased significantly compared with the control, from 45.7±0.4 to 54.8±1.1% for 9 μmol/l FB_1_. However, no change occurred in the number of cells in G0/G1 phase (26). It has been demonstrated that FB_1_ interferes with the G1/S phase checkpoint, which leads to changes in the cell cycle in animal experiments (27). The percentage of cells blocked in the G0/G1 phase of the cell cycle has been shown to be increased by FB_1_ in swine peripheral blood mononuclear cells (23). Thus, there are different effects on the cell cycle and apoptosis in different types of cell following treatment with FB_1_. The stimuli for cell proliferation in HEECs are a common result of the FB_1_-induced changes in the cell cycle and apoptosis. To further investigate the pathogenesis of the effects of FB_1_ in the present study, the protein expression of genes involved in the cell cycle in HEECs were analyzed using western blotting.

In eukaryotes, the cell cycle is tightly regulated by a number of protein kinases composed of CDKs, with corresponding regulatory cyclins and CDK inhibitors (28). The activity of the CDK/cyclin complexes is regulated by proliferating cell nuclear antigen, which binds to cyclin D1; cyclin E promotes the progression through G1 phase into S phase. The activity of the CDK/cyclin complexes is negatively regulated by binding to CDK inhibitors. CDK inhibitors are grouped into two distinct families (29): the INK4 family, including p15INK4b, p16INK4a, p18INK4c and p19INK4d (30), and the CIP/KIP family, including p21WAF1/CIP1, p27KIP1 and p57KIP2 (31). In the present study, the increased expression of cyclin D1 and decreased expression of CDK inhibitors, including p21 and p27, strongly suggest that FB1 induced the low expression of the members of KIP/CIP family, which sequentially stimulated the activities of the CDK/cyclin complexes.

In conclusion, the present *in vitro* study demonstrated that FB_1_ stimulated cell proliferation in HEECs, most likely by decreasing the percentage of cells in the G0/G1 phase of the cell cycle, increasing the percentage of cells in S phase, increasing the percentage of cells in G2/M phase and arresting cell apoptosis. The changes in the cell cycle may have been mediated by stimulation of cyclin D1 and inhibition of p21 and p27 expression, thereby accelerating the passing of cells through the G1-S checkpoint (32,33). The inhibition of cyclin E was offset by the role of these genes. It has been shown that the expression of cyclin D1, p21 and p27 is involved in the occurrence of esophageal cancer (34), and p21 and p27 have been proposed as candidate tumor suppressor genes (35). In a study by Huang *et al*(36), cyclin D1 was shown to be overexpressed in esophageal cancer in southern China (36). Furthermore, it has been demonstrated that the overexpression of cyclin D1, rather than cyclin E, is involved in the pathogenesis of esophageal cancer (37). However, additional studies, particularly *in vivo* experiments, are required to further demonstrate the effect of FB_1_ in normal human esophageal epithelium and to elucidate the correlation between FB_1_ and human esophageal cancer.

## Figures and Tables

**Table I:** Antibodies for western blot analysis.

Antibody	Source	Producer	Dilution
Cyclin D1	Rabbit polyclonal Ab	Cell Signaling Tech, USA	1:1,000
Cyclin E (HE12)	Mouse monoclonal Ab	Cell Signaling Tech, USA	1:1,000
p16 INK4A	Rabbit polyclonal Ab	Cell Signaling Tech, USA	1:1,000
p21 Waf1/Cip1 (DCS60)	Mouse monoclonal Ab	Cell Signaling Tech, USA	1:2,000
p27 Kip1 (SX53G8.5)	Mouse monoclonal Ab	Cell Signaling Tech, USA	1:1,000
Actin (AC-15)	Mouse monoclonal Ab	Sigma, USA	1:2,000
Mouse IgG, HRP-conjugated	Goat anti-mouse polyclonal Ab	KPL, UK	1:6,000
Rabbit IgG, HRP-conjugated	Goat anti-rabbit polyclonal Ab	Upstate, UK	1:6,000

IgG, immunoglobulin G; HRP, horseradish peroxidase; Ab, antibody; Cell Signaling Tech, Cell Signaling Technology, Inc.

## References

[1] Theumer MG, Clop EM, Rubinstein HR, Perillo MA (2008). The lipid-mediated hypothesis of fumonisin B1 toxicodynamics tested in model membranes. Colloids Surf B Biointerfaces.

[2] Stockmann-Juvala H, Savolainen K (2008). A review of the toxic effects and mechanisms of action of fumonisin B1. Hum Exp Toxicol.

[3] Cano-Sancho G, Ramos AJ, Marin S, Sanchis V (2012). Occurrence of fumonisins in Catalonia (Spain) and an exposure assessment of specific population groups. Food Addit Contam Part A Chem Anal Control Expo Risk Assess.

[4] van der Westhuizen L, Shephard GS, Rheeder JP, Burger HM (2010). Individual fumonisin exposure and sphingoid base levels in rural populations consuming maize in South Africa. Food Chem Toxicol.

[5] Alizadeh AM, Rohandel G, Roudbarmohammadi S (2012). Fumonisin B1 contamination of cereals and risk of esophageal cancer in a high risk area in northeastern Iran. Asian Pac J Cancer Prev.

[6] Sun G, Wang S, Hu X (2007). Fumonisin B1 contamination of home-grown corn in high-risk areas for esophageal and liver cancer in China. Food Addit Contam.

[7] Schwerdt G, Königs M, Holzinger H, Humpf HU, Gekle M (2009). Effects of the mycotoxin fumonisin B(1) on cell death in human kidney cells and human lung fibroblasts in primary culture. J Appl Toxicol.

[8] Galvano F, Russo A, Cardile V, Galvano G, Vanella A, Renis M (2002). DNA damage in human fibroblasts exposed to fumonisin B(1). Food Chem Toxicol.

[9] Seefelder W, Humpf HU, Schwerdt G, Freudinger R, Gekle M (2003). Induction of apoptosis in cultured human proximal tubule cells by fumonisins and fumonisin metabolites. Toxicol Appl Pharmacol.

[10] Kouadio JH, Mobio TA, Baudrimont I, Moukha S, Dano SD, Creppy EE (2005). Comparative study of cytotoxicity and oxidative stress induced by deoxynivalenol, zearalenone or fumonisin B1 in human intestinal cell line Caco-2. Toxicology.

[11] Bondy GS, Barker MG, Lombaert GA (2000). A comparison of clinical, histopathological and cell-cycle markers in rats receiving the fungal toxins fumonisin B1 or fumonisin B2 by intraperitoneal injection. Food Chem Toxicol.

[12] Wang SK, Liu S, Yang LG (2013). Effect of fumonisin B1 on the cell cycle of normal human liver cells. Mol Med Rep.

[13] Czerednik A, Busscher M, Bielen BA, Wolters-Arts M, de Maagd RA, Angenent GC (2012). Regulation of tomato fruit pericarp development by an interplay between CDKB and CDKA1 cell cycle genes. J Exp Bot.

[14] Yamamoto S, Kohsaka S, Nakajima K (2012). Role of cell cycle-associated proteins in microglial proliferation in the axotomized rat facial nucleus. Glia.

[15] Moreira PR, Guimarães MM, Guimarães AL (2009). Methylation of P16, P21, P27, RB1 and P53 genes in odontogenic keratocysts. J Oral Pathol Med.

[16] de Andrade BA, León JE, Carlos R, Delgado-Azañero W, Mosqueda-Taylor A, de Almeida OP (2012). Immunohistochemical expression of p16, p21, p27 and cyclin D1 in oral nevi and melanoma. Head Neck Pathol.

[17] Myburg RB, Dutton MF, Chuturgoon AA (2002). Cytotoxicity of fumonisin B1, diethylnitrosamine, and catechol on the SNO esophageal cancer cell line. Environ Health Perspect.

[18] Wang H, Wei H, Ma J, Luo X (2000). The fumonisin B1 content in corn from North China, a high-risk area of esophageal cancer. J Environ Pathol Toxicol Oncol.

[19] Severino L, Russo R, Luongo D, De Luna R, Ciarcia R, Rossi M (2008). Immune effects of four Fusarium-toxins (FB1, ZEA, NIV, DON) on the proliferation of Jurkat cells and porcine lymphocytes: in vitro study. Vet Res Commun.

[20] Taranu I, Marina DE, Burlacu R, Pinton P, Damian V, Oswald IP (2010). Comparative aspects of in vitro proliferation of human and porcine lymphocytes exposed to mycotoxins. Arch Anim Nutr.

[21] Gelderblom WC, Galendo D, Abel S, Swanevelder S, Marasas WF, Wild CP (2001). Cancer initiation by fumonisin B(1) in rat liver - role of cell proliferation. Cancer Lett.

[22] McKean C, Tang L, Tang M (2006). Comparative acute and combinative toxicity of aflatoxin B1 and fumonisin B1 in animals and human cells. Food Chem Toxicol.

[23] Marin DE, Gouze ME, Taranu I, Oswald IP (2007). Fumonisin B1 alters cell cycle progression and interleukin-2 synthesis in swine peripheral blood mononuclear cells. Mol Nutr Food Res.

[24] Fornelli F, Minervini F, Mulè G (2004). Cytotoxicity induced by nivalenol, deoxynivalenol, and fumonisin B1 in the SF-9 insect cell line. In Vitro Cell Dev Biol Anim.

[25] Seegers JC, Joubert AM, Panzer A (2000). Fumonisin B1 influenced the effects of arachidonic acid, prostaglandins E2 and A2 on cell cycle progression, apoptosis induction, tyrosine- and CDC2-kinase activity in oesophageal cancer cells. Prostaglandins Leukot Essent Fatty Acids.

[26] Mobio TA, Anane R, Baudrimont I (2000). Epigenetic properties of fumonisin B(1): cell cycle arrest and DNA base modification in C6 glioma cells. Toxicol Appl Pharmacol.

[27] Ramljak D, Calvert RJ, Wiesenfeld PW (2000). A potential mechanism for fumonisin B(1)-mediated hepatocarcinogenesis: cyclin D1 stabilization associated with activation of Akt and inhibition of GSK-3beta activity. Carcinogenesis.

[28] Canavese M, Santo L, Raje N (2012). Cyclin dependent kinases in cancer: Potential for therapeutic intervention. Cancer Biol Ther.

[29] Douville JM, Cheung DY, Herbert KL, Moffatt T, Wigle JT (2011). Mechanisms of MEOX1 and MEOX2 regulation of the cyclin dependent kinase inhibitors p21 and p16 in vascular endothelial cells. PLoS One.

[30] Canepa ET, Scassa ME, Ceruti JM (2007). INK4 proteins, a family of mammalian CDK inhibitors with novel biological functions. IUBMB Life.

[31] Ito Y, Yoshida H, Matsuzuka F (2004). Expression of the components of the Cip/Kip family in malignant lymphoma of the thyroid. Pathobiology.

[32] Sugimoto M, Martin N, Wilks DP (2002). Activation of cyclin D1-kinase in murine fibroblasts lacking both p21(Cip1) and p27(Kip1). Oncogene.

[33] Verlinden L, Verstuyf A, Convents R, Marcelis S, Van Camp M, Bouillon R (1998). Action of 1,25(OH)_2_D3 on the cell cycle genes, cyclin D1, p21 and p27 in MCF-7 cells. Mol Cell Endocrinol.

[34] Hirai T, Kuwahara M, Yoshida K, Osaki A, Toge T (1999). The prognostic significance of p53, p21 (Waf1/Cip1), and cyclin D1 protein expression in esophageal cancer patients. Anticancer Res.

[35] Faria MH, Patrocinio RM, Moraes Filho MO, Rabenhorst SH (2007). Immunoexpression of tumor suppressor genes p53, p21 WAF1/CIP1 and p27 KIP1 in human astrocystic tumors. Arq Neuropsiquiatr.

[36] Huang XP, Rong TH, Lin P (2006). Cyclin D1 overexpression in esophageal cancer from southern China and its clinical significance. Cancer Lett.

[37] Anayama T, Furihata M, Takeuchi T (2001). Insufficient effect of p27(KIP1) to inhibit cyclin D1 in human esophageal cancer in vitro. Int J Oncol.

